# Combined Effect of Genetic Variants on Long-Term Weight Response after Bariatric Surgery

**DOI:** 10.3390/jcm12134288

**Published:** 2023-06-26

**Authors:** Macarena Torrego-Ellacuría, Ana Barabash, Pilar Matía-Martín, Andrés Sánchez-Pernaute, Antonio J. Torres, Alfonso L. Calle-Pascual, Miguel A. Rubio-Herrera

**Affiliations:** 1 Department of Endocrinology and Nutrition, Hospital Clínico San Carlos, IdISSC, 28040 Madrid, Spain; macarena.torrego@gmail.com (M.T.-E.);; 2 Faculty of Medicine, Department of Medicine, Universidad Complutense, 28040 Madrid, Spain; 3 Centro de Investigación Biomédica en Red de Diabetes y Enfermedades Metabólicas Asociadas (CIBERDEM), 28040 Madrid, Spain; 4 Department of Surgery, Hospital Clínico San Carlos, IdISSC, 28040 Madrid, Spain

**Keywords:** risk score, SNP, bariatric surgery, weight regain, weight loss

## Abstract

The pathophysiology of body weight control involves complex interactions between hormonal, environmental, behavioral and genetic factors. The purpose of this study was to analyze the association between single nucleotide polymorphisms (SNPs) of 13 genes encoding gastrointestinal peptides, their receptors or the proteins involved in their expression, with long-term weight response in a cohort of 375 patients undergoing bariatric surgery (BS). To evaluate weight response, we combined several variables to define specific response phenotypes six years after surgery. The study protocol was registered in ISRCTN (ID80961259). The analysis of the selected SNPs was performed via allelic discrimination using Taqman^®^ probes (Applied Biosystems, Foster City, CA, USA). The genotype association study was performed using the SNPstat program, with comparisons adjusted for sex, age, initial body mass index, type 2 diabetes, hypertension diagnosis and the type of surgery. We identified eight genetic variants associated with the weight response to BS, independently of the presurgery patient profile and the type of surgical technique, from which we calculated the unweighted risk score (RS) for each phenotype. The highest scoring category in each RS was significantly associated with lower weight loss (*p* = 0.0001) and greater weight regain (*p* = 0.0012) at the end of the follow-up.

## 1. Introduction

Bariatric surgery (BS) achieves substantial and persistent effects on weight loss in patients with morbid obesity (MO) and improves the management of obesity-associated comorbidities [1,2,3,4]. However, there is inter-individual variability in terms of the maximum weight loss achieved [5,6] and long-term weight regain [7,8]. Genetic variation among individuals underlies the variety of physiological responses in the context of BS, caloric restriction and altered gastrointestinal hormones [9], with a lower degree of variability in weight loss observed in genetically related subjects compared to genetically unrelated matched individuals in the medium to long term after the intervention [10]. 

Genome-wide association studies (GWAS) have identified more than 300 single nucleotide polymorphism (SNP) involved in eating behavior and energy expenditure, associated with body mass index (BMI) and adiposity traits [11,12,13]; however, in BS intervention, studies are very limited [14]. Since 2003, Genetic Risk Score (GRS) studies have been conducted to study weight loss and BMI evolution after BS, based on groups of SNPs in loci identified in previous GWASs and replicated in various populations [12,15,16,17]. Depending on the study, the SNPs included Vary. The results of the GRS studies suggest that some variants may modulate differences in weight response to BS, but there is little evidence generated on the subject.

The primary objective of our work was to identify genetic markers associated with weight loss and its long-term maintenance after different BS surgical techniques. Considering the physiological mechanisms involving gastrointestinal peptides and their signaling in the appetite-regulating brain nuclei involved in body weight control at gastrointestinal and hypothalamic level [18,19], the genetic variants selected encode for peptides, their receptors or the proteins involved in their expression that are implicated in the control of energy intake and expenditure.

## 2. Materials and Methods

This is a single-center retrospective study based on a prospective database from the Hospital Clinico San Carlos, Madrid (HCSC). The cohort included 375 patients aged between 18 and 65 years (BMI > 40 kg/m^2^ or BMI ≥ 35 kg/m^2^ associated with comorbidity) that were selected from a cohort of 510 subjects who underwent a first bariatric surgical procedure between 2009 and 2014, after applying the exclusion criteria previously reported [20] together with the exclusion of subjects of Latin ethnicity and without genotyping. The project was approved by the HCSC Clinical Research Ethics Committee (16 February 2009). The study protocol was registered at https://www.isrctn.com/ (accessed on 1 January 2023) (ID ISRCTN80961259). Demographic, clinical and anthropometric information was collected in the electronic health records prior to surgery. Type 2 diabetes (T2D) and hypertension (HTN) were diagnosed and categorized. The different surgical techniques (STs) were sleeve gastrectomy (SG), Roux-en-Y gastric bypass (RYGB), biliopancreatic diversion with or without duodenal switch (BPD-DS) and single anastomosis duodeno-ileal bypass with sleeve gastrectomy (SADI-S), as malabsorptive procedures. The type of surgical technique was chosen according to clinical practice criteria of hospital protocol based on age, BMI and comorbidities. 

The 375 cases were followed up after surgery, with annual appointments up to 8 years with weight measurements [20]. The main variables for assessing weight response include the percentage of total weight lost (%TWL), the percentage of excess weight loss (%EWL), with ideal weight calculated for a BMI of 25 kg/m^2^ and WR as a percentage of maximum weight loss (%WR_MWL) [21]. Nadir weight was determined based on all the postoperative weight measures available, considering the lowest value. The end of clinical follow-up was established in year 6 since it was the common period of follow-up of the entire sample according to the inclusion dates. 

### 2.1. Selection of Candidate SNPs 

The genetic variants included in the association study with weight response were 48 SNP-like variants of 13 genes: GHSR, WFS1, BDNF, MC4R, GIPR, DPPIV, NPYR, CLOCK, GLP1R, TCF7L2, KCNJ11, FTO and PYY. Table 1 lists the genes and SNPs included, with the reference allele in each category. The genetic variants of the CLOCK gene (rs3749474, rs1801260 and rs4580704) were analyzed in a previous work undertaken by our group [22]. 

The genetic variants included were chosen following the candidate gene selection strategy. On the one hand, the SNPs of genes, which have been previously described as being associated with obesity or weight response phenotypes, or which have functional repercussions (modification of protein expression or structure), were studied directly (MC4R, FTO, BDNF, GHSR, WFS1, GIPR, TCF7L2, KCNJ11 and CLOCK genes). For candidate genes where no associated phenotype has yet been described (GLP1R, DPPIV, NPYR and PYY genes), a haplotype study was performed using a tagSNP approach. The selection of the tag SNPs (minimum allele frequency MAF—greater than 5%) was carried out by consulting public-domain specialized databases for each candidate gene (HapMap International public project [23], dbSNP [24] and Ensembl project [25]). To identify the tag SNPs, the Haploview 4.1 program was used, which handles the data from the aforementioned databases. We included tag SNPs that allow for the detection of haplotypes with a frequency higher than 10% in the Caucasian population.

### 2.2. DNA Extraction and Genotyping of Samples

Two peripheral blood tubes (EDTA) of 10 mL each were collected from each patient. DNA was extracted from peripheral leukocytes after a series of washes with red cell lysis buffer and further treated with the DNAzol^®^—Genomic DNA Isolation Reagent extraction kit, following the manufacturer’s protocol. The concentration and purity were determined using a NanoDrop™ 2000/2000C spectrophotometer. Genotyping was performed using predesigned TaqMan assays for each SNP (Assay ID included in Table 1) using a 7500 Fast Real-Time PCR System (Applied Biosystems, Foster City, CA, USA). A genotyping call rate over 95% per plate, negative sample controls and three well-differentiated genotyping clusters were required to validate results.

### 2.3. Statistical Analyses

The data were expressed as the mean and standard deviation or median and interquartile range for continuous variables and absolute and relative frequencies for categorical variables. SNPStats software was used to evaluate Hardy–Weinberg equilibrium and the genotype association study with weight response at nadir and at year 6, under multiple inheritance models with respect to allele reference homozygotes [24]: co-dominant, dominant, recessive, over-dominant and log-additive [26]. To evaluate the best model, the Akaike Information Criterion (AIC) was used, choosing the model with the lowest AIC value. A linear regression analysis was performed for quantitative response variables (%TWL, %EWL and %WR_MWL), expressing the results with the mean, standard error and mean differences (95%CI). For response variables coded as a binary variable (%WR_MWL > 20% [21], %EWL > 50% [27]), a logistic regression analysis was performed, expressing the results including genotype frequencies, proportions and OR (95%CI).

The association study of SNPs and weight loss and regain after BS was performed on the overall sample. With the results of the association of each individual variant with the weight response to BS, an unweighted risk score (RS) was calculated for each associated phenotype: RS_%TWL_nadir, RS_%TWL_6y, and RS-%WR_MWL. Each SNP was assigned a value of 2 for the homozygote of the risk allele, a value of 1 for the heterozygote of the risk allele, and a value of 0 for all other combinations. We refer to the risk allele for each SNP as the one that was significantly associated with lower weight loss or higher weight regain in the association study in our cohort. The sum of the genetic variant scores was calculated to obtain the RS-associated phenotype (RS-phenotype) scoring per patient. The RS- phenotype scoring was coded as a categorical variable around the 75th percentile (score P > 75 vs ≤P75). The association of the RS-phenotype codified scoring categories with the weight response variables was studied by means of logistic or linear regression analyses, replicating the SNPS-weight response association analysis. All of the comparisons were adjusted for sex, age, initial BMI, pre-surgery T2D and HTN diagnosis and the type of surgery. All *p*-values lower than 0.05 were deemed statistically significant.

## 3. Results

### 3.1. Association Study with Weight Response

The percentage of surgical techniques performed in these cases were as follows, 16% SG, 54.66% RYGB, and 29.3% malabsorptive (77% SADI-S, 23% BPD-DS), with a median follow-up of six years (IQR = 5–8) after BS. At the time of the surgery, the mean BMI was 44.87 ± 6.59 kg/m^2^, and the prevalence of T2D and HTN was 35.7% and 49%, respectively. Table 2 shows the description of the demographic profile and the weight loss and weight regain variables included as phenotypes in the association study.

**Figure 1:**
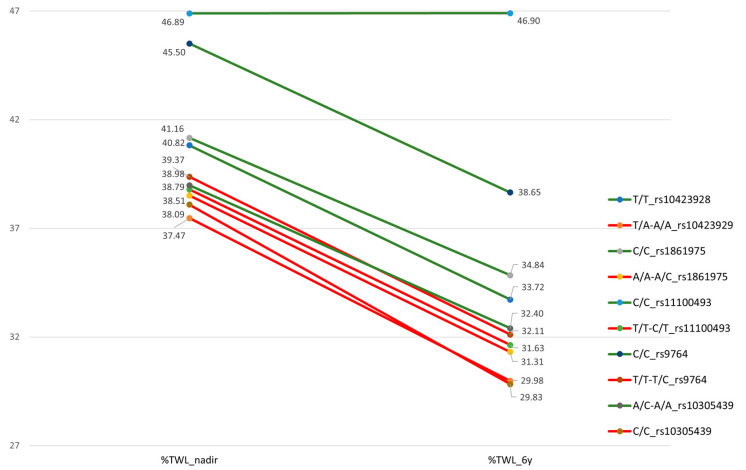
Mean values of %TWL at nadir and at year 6 according to the genetic variants.

From the 48 SNPs analyzed, a total of eight showed an association with weight response after BS after adjusting for age, sex, T2D, HTN, type of surgical technique and initial BMI; five variants showed an association with weight loss and three variants with weight regain. Figure 1 illustrates the mean values of %TWL at nadir and at the end of the follow-up of the variants with significative association according to dominant or recessive model, differentiating for each SNP, the genotype with a risk allele shown in red font. The variant rs10423928 of the GIPR gene and rs1861975 of the DPPIV gene showed associations with %TWL at nadir and at year 6. The variant rs9764 of NPY1R showed an association with %TWL at nadir. The variants rs11100493 of NPY5R, rs1801260 of the CLOCK gene and rs10305439 and rs2143734 of the GLP1R gene showed associations with %TWL at the end of the follow up. The variant rs1801260 of the CLOCK gene, rs10305439 and rs877446 of the GLP1R gene showed significant association with %WR_MWL at the end of the follow up. The mean differences achieved (IC95%; *p*) are described in Table 3. AIC values for each model in the genotype association study are included in Supplementary Table S1.

TWL, total weight loss. Red lines: risk genotypes, associated with lower %TWL.

With the %EWL phenotype, no variants showed significant association with %EWL at nadir. The number of variants associated with %EWL at the end of the follow-up was reduced to 2, with the same sense of association and varying quantitative differences with respect to %TWL: SNP rs1861975 of DPPIV gene [mean difference %EWL (IC95%) = 10.33 (1.79–18.88); *p* = 0.018]; SNP rs10305439 of the GLP1R gene [mean difference %EWL (IC95%) = 6.45 (0.91–12.00); *p* = 0.023].

The remaining variants showed no significant association with any of the weight response phenotypes analyzed.

### 3.2. Clustered Risk and Weight Response

Once the risk alleles for each genetic variant and weight response phenotype were identified, three RS-phenotypes (RS_%TWL_nadir, RS_%TWL_6y and RS-%WR_MWL) were calculated. The results of the logistic and linear regression analysis of the RS-phenotype scoring categories and the variables %TWL_nadir, %TWL_6y and %WR_MWL are shown below. All of the results were adjusted by age, sex, T2D, HTN, type of surgical technique and initial BMI. The mean score of the RS_%TWL_nadir was 2.5 ± 1.28 points (0–6 points). No association between RS_%TWL_nadir and %TWL_nadir was found.

**Figure 2:**
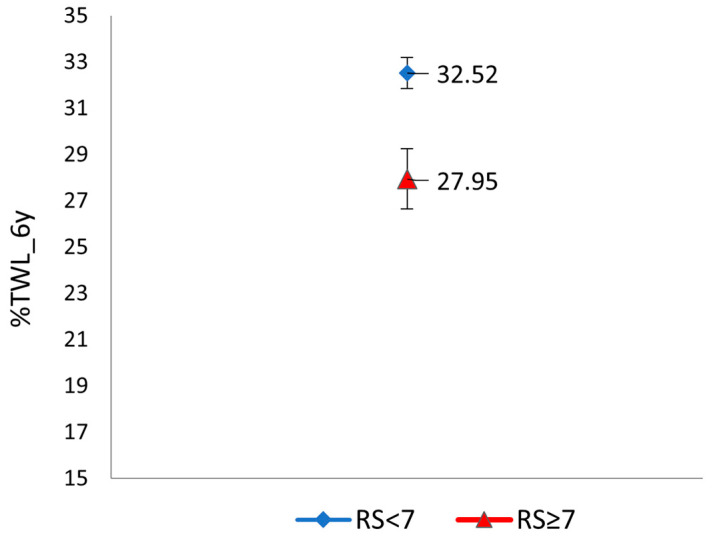
Mean %TWL at year 6 according to RS_%TWL_6y scoring categories.

The mean score of RS_%TWL_6y was 4.86 ± 1.66 points (0–12 points). A score of ≥7 points was significantly associated with weight loss at the end of the follow-up (Figure 2), with a mean difference in terms of %TWL_6y [Mean difference (IC95%) = −5.37 (−7.97–−3.62); *p* = 0.0001]. Scoring ≥ 7 points was also associated with a significant risk of achieving a %EWL > 50% [OR (95%CI) = 0.46 (0.23–0.90); *p* = 0.026]. Table 4 includes a comparison of the patient profile according to the RS_%TWL_6y scoring categories. No significant differences were found in any of the variables included.

The mean RS-%WR_MWL score was 3.42 ± 1.46 points (0–12 points). A score of ≥7 points was significantly associated with weight regain, with a mean difference in %WR_MWL [mean difference (IC95%) = 7.06 (2.80–11.31); *p* = 0.0012] and with a significant risk of achieving an %WR_MWL > 20% [OR (95%CI) = 2.01 (1.22–3.31); *p* = 0.0059].

## 4. Discussion

In our work, the combined effect of genetic variants of GIPR, DPPIV, NPY1R, NPY5R, CLOCK, and GLP1R genes was significantly associated with weight loss and long-term weight regain. The risk score for the associated phenotype %TWL at year 6 included six SNPs, of which only the rs9939973 variant of the FTO gene has been included in previous GRS studies [28]. The RS_%TWL_6y scoring category of seven points or more, which accounts for 18% of the sample, was associated with a mean %TWL at the end of follow-up 5.37 times lower than the mean %TWL of subjects scoring less than seven points (*p* = 0.0001) and 2.17 times more likely to achieve an %EWL less than 50% (*p* = 0.026). The weight loss achieved and maintained at year 6 was similar or even superior to previous studies [29,30,31] that include a long-term follow-up [32], with 83% of the sample reaching an EWL above 50% at the end of the follow-up.

Most published studies conclude that weight loss appears to be influenced by multiple genetic variants, which interact with each other and with phenotypic traits. The results of the review by Gupta et al. [33] demonstrate that the combination of several genes, as measured by genetic risk scores (GRS) in various studies [15,34,35] may have significant predictive value after surgery. Genetic variants included in the GRS for weight development after BS usually include hypothalamic genes related to monogenic obesity, involved in the regulation of energy homeostasis, mainly the hypothalamic leptin-melanocortin system [36]. In the study by Rinella et al., 17 SNPs with potential clinical utility were identified from the 111 gene variants included, and the combined association with weight loss after BPGYR was studied [37]. De Toro et al. studied the combined effect of 186 SNPs with a polygenic risk score model in patients undergoing biliopancreatic diversion with duodenal switch; however, only 11 variants showed a significant association with %EWL [35]. In the OBEGEN study, a clinical-genetic predictive model of response was obtained by combining three clinical variables (age, type of surgery and the presence of T2D) and nine SNPs out of the fifty analyzed [38]. It should be noted that in all these studies, the ratio of variants with significant associations with weight loss with respect to those included in the methodology ranges between 6 and 20%. Moreover, due to methodological differences in the calculation of the risk score, follow-up after BS, types of surgical techniques and adjustment for covariables performed, no conclusive results concerning the key variants to be included in the GRS models can be obtained.

The phenotype of weight response was %EWL in most studies, with a follow-up of up to four years after BS. In our study, a follow-up of six years, considered long-term, was carried out, and weight regain phenotypes were included. Weight regain measured with respect to maximum weight loss achieved was lower than in previous studies [21,39]. The results of the association study showed that a greater number of significant associations were found with the variable %TWL as a phenotype than with %EWL. Previous findings of the original cohort showed that %TWL enabled better differentiation of weight loss trajectories by surgical technique, age, sex and comorbidities [20]. Other studies suggest that %TWL is the consistent measure for comparing weight loss between cohorts [40,41] as it allows better averaging of individual weight response without reference to ideal BMI.

Our work includes 5 of the 39 genes included in the study by Ciudin et al. [38], together with variants not studied in previous studies concerning risk scores. The 48 SNPs included were chosen for their involvement in the pathophysiology of weight control, at the gastrointestinal or hypothalamic levels [19,42]. There are multiple neuronal circuits involved in the control of appetite regulation and energy expenditure [43,44], which encode neuropeptides synthesized in central and peripheral neurons, together with the endocrine cells of the gastrointestinal tract and other endocrinologically active organs [43,45]. The genetic variants with significant association from GIPR, DPPIV, NPY1R, NPY5R and GLP1R genes included in the risk scores are related to these neural axis circuits.

In the methodology for calculating the RS-associated phenotype, we used an unweighted model previously described in other studies [14,34,38], assigning risk scoring according to the combination of risk alleles of the SNPs identified in the study of the association of each individual variant with weight response. Adjustment variables include potential predictors of long-term weight regain identified in a previous analysis by our group based on the original cohort [20]. As a phenotype for calculating the risk score in our series, we used the quantitative variable %TWL and %WR with respect to the maximum weight loss achieved without establishing a cut-off point, since there is no defined phenotype nor a defined cut-off point [32] for long-term weight response after BS. The %EWL variable with a cut-off point of 50% is the criterion used in most of the GRS studies [14,34,35,37,38]; although in the short term after BS, so it follows that the most significant results with weight response have been obtained when using an extreme phenotype of weight loss. The association found in our sample with this coded phenotype would allow us to categorize the patients of our cohort as “hyper-responder” or “hypo-responder”, according to Bonouvrie et al. [27].

The higher magnitude of statistical significance found in our sample when combining several SNPs, pooling risk alleles for a given phenotype is consistent with what has been reported in the literature: weight loss after BS may be influenced by multiple genetic variants that have modest individual effects, but synergistically produce a larger aggregate effect. As such, polygenic risk scores may better capture the genetic architecture of weight loss with BS [33].

Some limitations related to long-term response to BS must be taken into consideration, such as the lack of information on variables with potential impact on weight evolution such as dietary intake and behavior, hormonal disturbances, weight loss medication and the level of physical activity. Adjustments made based on the clinical profile and BMI at the time of the surgery minimize the effect of potential confounding factors. Moreover, the clinical profile of the patient when stratifying according to SR scoring categories was comparable. The selection of genetic variants may miss potential SNPs with an effect on the weight response, for which there is currently no proven evidence. Despite these limitations, our strengths include a high rate of patient retention throughout a long follow-up and having included phenotypes for both weight loss and regain.

In summary, in this large cohort of patients followed up to six years we have identified genetic variants with combined effect in the weight response, some of which have not previously been described. The aggregation of SNPs associated with weight loss and regain in our sample was different, suggesting a distinct grouped risk combination by weight response phenotype.

## Figures and Tables

**Table 1:** Genes and SNP included in the association study.

Candidate Gen	SNP	Reference Allele	Assay ID
GHSR	rs572169	C	C___1079489_20
WFS1	rs10010131	A	C__30473796_10
BDNF	rs6265	C	C__11592758_10
MC4R	rs17782313	T	C__32667060_10
GIPR	rs10423928	T	C__30103605_10
DPPIV	rs17759529	C	C__34245343_10
DPPIV	rs2389643	C	C__15784426_10
DPPIV	rs2268889	C	C__15875589_10
DPPIV	rs12995983	T	C___2789708_20
DPPIV	rs3788979	C	C___2789710_10
DPPIV	rs741529	G	C___2789719_10
DPPIV	rs12469968	G	C___2789726_10
DPPIV	rs1861975	A	C___2789730_10
NPY2R	rs6849115	T	C__30852111_10
NPY2R	rs11099992	A	C_____44829_10
NPY2R	rs6857715	C	C__29013142_10
NPY2R	rs1047214	C	C___7427258_20
NPY2R	rs17304901	G	C__32705343_10
NPY2R	rs11728843	G	C__30852113_10
NPY1R	rs9764	T	C___8788046_10
NPY1R	rs7687423	A	C___8066900_10
NPY1R	rs11100489	T	C__31208177_10
NPY5R	rs11100493	T	C_____74249_10
NPY5R	rs4632602	C	C__29684077_20
NPY5R	rs11724320	T	C_____74248_10
NPY5R	rs7678265	C	C_____74246_30
CLOCK	rs3749474	C	C__26405955_10
CLOCK	rs1801260	A	C___8746719_20
CLOCK	rs4580704	G	C__28028791_10
GLP1R	rs10305439	C	C___2491169_10
GLP1R	rs2143734	A	C__16072581_20
GLP1R	rs877446	A	C__11607361_10
GLP1R	rs6923761	G	C__25615272_20
GLP1R	rs932443	T	C___2491141_10
GLP1R	rs2300612	T	C__15755173_10
GLP1R	rs2268640	G	C___2491124_10
TCF7L2	rs7903146	C	C__29347861_10
TCF7L2	rs12255372	G	C____291484_20
KCNJ11	rs5215	C	C___2991148_10
KCNJ11	rs5218	G	C___2991149_20
KCNJ11	rs5219	T	C__11654065_10
KCNJ11	rs886288	A	C___9686373_10
FTO	rs9939609	T	C__30090620_10
FTO	rs9939973	G	C__11776771_10
PYY	rs2700831	T	C___2964503_10
PYY	rs9890045	G	C__30502516_20
PYY	rs1684668	T	C__11887233_10
PYY	rs1618809	A	C__27061985_10

**Table 2:** Demographic profile and weight response variables at the follow-up. (N = 375).

Variable	Value
Age, in years	44.79 ± 11.99
Female gender, n (%)	259 (69)
%TWL_nadir,	38.79 ± 9.84
%EWL_nadir,	91.19 ± 23.69
%TWL_6y	31.67 ± 11.62
%EWL_6y	74.08 ± 26.89
%EWL6y > 50%, n (%)	311 (82.93)
%WR_MWL, median (IQR)	15.76 (7.99–28.69)
%WR_MWL > 20%, n (%)	154 (41.1)

Mean (SD) unless otherwise stated. TWL, total weight loss; EWL, excess weight loss; nadir, maximum weight loss achieved; 6y, at 6 years of follow-up; WR_MWL, percentage of weight regain from the maximum weight loss.

**Table 3:** Variants with significant association with weight response: Mean differences between genotypes (N = 375).

Gene	SNP	%TWL_nadir	%TWL_6y	%WR_MWL	Risk Allele
GIPR	10423928	−3.32 (−5.53–−1.11); 0.0036	−3.55 (−6.33–−0.77); 0.013		A
DPPIV	1861975	2.92 (−0.11–5.72); 0.042	3.92 (0.47–7.36); 0.026		A
NPY1R	9764	4.61 (0.09–9.13); 0.047			T
NPY5R	11100493		22.20 (2.19–42.20); 0.03		T
CLOCK	1801260		1.85 (0.07–3.62); 0.042	−3.27 (−6.42–−0.12); 0.042	A
GLP1R	10305439		2,71 (0.41–5.01); 0.022	−4.88 (−8.84–−0.92); 0.016	C
GLP1R	2143734		−1.76 (−3.46–−0.05); 0.039		G
GLP1R	877446			−5.03 (−9.72–0.34); 0.036	A

Mean difference (IC95%); *p*. TWL, total weight loss; %TWL_nadir, percentage of total weight loss at nadir; %TWL_6y, percentage of total weight loss at year 6; %WR_MWL, percentage of weight regain from the maximum weight loss to year 6. Results adjusted by age, sex, T2D, HTN, type of surgical technique and initial BMI.

**Table 4:** Comparative pre-surgery variables according to RS_%TWL_6y scoring categories.

	RS_%TWL_6y Categories		
Variable	RS < 7 N = 306	RS ≥ 7 N = 70	*p*
BMI_00, kg/m^2^	44.65 (6.50)	45.77 (6.98)	0.225
Age, in years	44.79 (12.25)	44.77 (10.86)	0.988
Female gender, n (%)	212 (69.51)	47 (67.1)	0.73
T2D, n (%)	108 (35.4)	26 (37.1)	0.77
HTN, n (%)	141 (46.22)	41 (58.57)	0.06
Restrictive n (%)	52 (17.05)	8 (11.42)	0.25
Mixed, n (%)	164 (53.77)	41 (58.57)	0.45
Malabsorptive, n (%)	89 (29.18)	21 (30)	0.87

Mean (SD) unless otherwise stated. BMI_00, pre-surgery body mass index; T2D, type 2 diabetes; HTN, hypertension; RS, risk score; RS_%TWL_6y, risk score for total weight loss at year 6.

## Data Availability

Not applicable.

## Supplementary Materials

The following supporting information can be downloaded at: https://www.mdpi.com/article/10.3390/jcm12134288/s1, Table S1: Genotype association study: Akaike Information Criterion (AIC) value for each model. Variants with significant association with weight response variables.
