# Supplementary material for: Combined Effect of Genetic Variants on Long-Term Weight Response after Bariatric Surgery
Source: J Clin Med. 2023 Jun 26;12(13):4288. doi: 10.3390/jcm12134288 (PMC10342460; doi:10.3390/jcm12134288)
Supplement: Supplementary file 1 [file jcm-12-04288-s001.zip › jcm-2398394-supplementary.pdf]

Supplementary Material

Table S1. Genotype association study: Akaike Information Criterion (AIC) value for each model. Variants with significant association with weight response variables.

**Association**		**AIC value for each model**			
**Gene**	**SNP-Phenotype**	**Co-dominant**	**Dominant**	**Recessive**	**Log-additive**
GIPR	10423928 - %TWL_nadir	1814.2	**1812.7**	1821.5	1814.6
	10423928 - %TWL_6y	1929.7	**1928.1**	1934.5	1929.5
DPPIV	1861975 -%TWL_nadir	2611.9	2614.4	**2610.2**	2613.6
	1861975 - %TWL_6y	2764.7	2767.6	**2762.8**	2765.9
NPY1R	9764 -%TWL_nadir	1804.5	1806.5	**1802.5**	1805.5
NPY5R	11100493 - %TWL_6y	2768.5	2771.9	**2767.5**	2772.3
CLOCK	1801260 - %TWL_6y	2808.7	2809	2808.2	**2807.4**
	1801260 - %WR_MWL	3239.6	3238.4	3240	**3237.6**
GLP1R	10305439 - %TWL_6y	2771.6	**2771.2**	2776.7	2775.7
	10305439 - %WR_MWL	3170.1	**3170**	3175.7	3174.3
	2143734 - %TWL_6y	2797.2	2797.1	2796.7	**2795.5**
	877446 - %WR_MWL	3222.5	**3220.7**	3224.5	3223

AIC, Akaike Information Criterion. In bold highlighted the AIC with the lowest value for each association.
